# Mucosal immune responses to *Ichthyophthirius multifiliis* in the ocular mucosa of rainbow trout (*Oncorhynchus mykiss*, Walbaum), an ancient teleost fish

**DOI:** 10.1007/s42995-023-00199-6

**Published:** 2023-10-31

**Authors:** Weiguang Kong, Guangyi Ding, Gaofeng Cheng, Peng Yang, Zhen Xu

**Affiliations:** 1 Key Laboratory of Breeding Biotechnology and Sustainable Aquaculture, Institute of Hydrobiology, Chinese Academy of Sciences, Wuhan, 430072 China; 2 Department of Aquatic Animal Medicine, College of Fisheries, Huazhong Agricultural University, Wuhan, 430070 China

**Keywords:** Ocular mucosa, Mucosal immunity, Transcriptome analysis, *Oncorhynchus mykiss*, *Ichthyophthirius multifiliis*

## Abstract

The eye, as a specialized visual organ, is directly exposed to the external environment, and, therefore, it faces constant challenges from external pathogenic organisms and toxins. In the ocular mucosa (OM) of mammals, mucosal-associated lymphoid tissues (MALTs) constitute the primary line of defense. However, the immune defense role of the OM remains unknown in aquatic vertebrates. To gain insights into the immune processes within the OM of teleost fish, we developed an infection model of rainbow trout (*Oncorhynchus mykiss*) OM using a parasite, *Ichthyophthirius multifiliis* (Ich). Immunofluorescence, qPCR, and H&E staining revealed that Ich successfully infiltrates the OM of rainbow trout, leading to pathological structural changes, as evidenced by A&B staining. Importantly, the qPCR results indicate an up-regulation of immune-related genes following Ich infection in the OM. Moreover, transcriptome analyses were conducted to detect immune responses and impairments in eye function within the OM of rainbow trout with Ich infection. The results of the transcriptome analysis that Ich infection can cause an extensive immune response in the OM, ultimately affecting ocular function. To the best of our knowledge, our findings represent for the first time that the teleost OM could act as an invasion site for parasites and trigger a strong mucosal immune response to parasitic infection.

**Supplementary Information:**

The online version contains supplementary material available at 10.1007/s42995-023-00199-6.

## Introduction

The eye, as a vital visual organ, gives vertebrates access to information from the outside environment (Baden et al. 2020; McGilligan et al. 2013). Notably, direct exposure of the eye to the external environment renders its ocular mucosa (OM) susceptible to invasion by pathogenic organisms. To counter these environmental insults, the vertebrate eye has a diverse repertoire of strategies to preserve visual function. These include anatomic and physical barriers, chemical factors within the tears, and innate and adaptive immune cells located in the tissue (Akpek and Gottsch 2003; McClellan 1997; Raposo et al. 2020). In mammals, eyelids covering the ocular surface are considered the first line of defense to the eye, which serves as a physical barrier between the inner eye tissue and external pathogens (Ansari 2016; Tawfik et al. 2016). Human eyelids feature a row of eyelashes along the eyelid margin that keep dust and foreign bodies away from the eye by causing the person to blink reflexively at the slightest provocation (de Paiva et al. 2022; Sridhar 2018). Moreover, the tear film contains abundant lysozyme, antimicrobial peptides, and immunoglobulins that can also protect the eye effectively (Azkargorta et al. 2017; McDermott 2013; McKay et al. 2020; Raposo et al. 2020). In contrast to mammals, teleost fish lack eyelashes, mobile eyelids, and lacrimal glands (Lamb et al. 2007, 2008), underscoring the heightened significance of OM immune function.

In mammals, the ocular surface is considered a mucosal tissue supported by a dedicated mucosal immune system (Ambroziak et al. 2016). The OM consists of an epithelium and lamina propria and contains different cell types for innate immune (macrophages and mast cells) and adaptive immunity (T and B lymphocytes) (de Paiva et al. 2022; del Palomar et al. 2019; Mircheff et al. 2005; Pennington et al. 2019; Reinoso et al. 2012). In birds and mammals, lymphocytes that reside within the ocular surface form a mucosal-associated lymphoid tissue (MALT) as eye-associated lymphoid tissue (EALT) provides an effective immune barrier between the OM and external environment (Knop and Knop 2000; van Ginkel et al. 2012). Teleost fish, possess a specialized mucosal innate and adaptive immune system. Teleost fish, as the most ancient bony vertebrates, exhibit diffuse mucosal-associated lymphoid tissues (MALTs) within their body surfaces (Gomez et al. 2013; Salinas et al. 2021), which could mount strong innate and adaptive immune responses upon parasitic infection (Garcia et al. 2022; Xu et al. 2013; Zhang et al. 2021). Due to living in water, teleost fish OM may be subjected to various pathogens and evolutionary selective force. Therefore, a mucosal immune response is indispensable for protecting the extensive and vulnerable mucosal surface. Meanwhile, it should be noted that strong mucosal immunity comes at a cost, and uncontrolled inflammatory responses may lead to blindness (Galletti et al. 2017). Dry eye disease (DED) produces inflammatory damage to the mucosal surface of the eye, which in severe cases can lead to significant discomfort, visual impairment, and blindness (Oganov et al. 2023; Perez et al. 2023). To maintain mucosal homeostasis, the mucosal immune system needs to decide whether to ignore the invasion of pathogenic microorganisms or initiate a massive inflammatory response (Galletti et al. 2017; Mircheff et al. 2005; Stern et al. 2010). However, whether teleost fish have evolved an effective OM immune system, and how the OM immune system functions to protect their ocular surface is an enigmatic question.

In this study, we utilized transcriptome analysis to investigate the interaction between the teleost OM and the parasite *Ichthyophthirius multifiliis* (Ich), which poses a significant threat to the global trout farming industry because of its potential to cause severe economic losses (Yang et al. 2023). Our findings highlight the successful invasion of the OM by Ich and the resulting pathological damage. Moreover, the presence of Ich triggers a local immune response within the OM, with both innate and acquired immunity contributing significantly in the early and late phases of infection, respectively. Additionally, Ich infection caused substantial downregulation of genes linked to eye development and visual perception in rainbow trout, resulting in impaired eye function. These results provide the first evidence of innate and adaptive immune responses within the OM of teleost fish following parasitic infection.

## Materials and methods

### Experimental animals

Rainbow trout (*Oncorhynchus mykiss*) (10–15 g) were purchased from a fish farm in Shiyan (Hubei, China), and kept in a 16 °C recirculating aquaculture system for two weeks. Before experimentation, the fish were acclimatized for a week and verified to be clinically healthy. Animal procedures were approved by the Animal Experiment Committee of the Institute of Hydrobiology, Chinese Academy of Sciences, and carried out according to the relative guidelines.

### Ich infection

The methods used for parasite isolation and infection were described previously by Xu et al*.* with slight modifications (Xu et al. 2013; Yu et al. 2018). Briefly, rainbow trout severely infected with Ich were euthanized with an overdose of MS-222, and the trophonts were taken out from the skin and fins with a clean cell scraper. After cleaning three times, the clean trophonts and tomonts were placed into a petri dish with sterile water to allow theront release; these were collected for experimental infection ~ 24 h later. For the infection, fish were exposed to an optimal single dose of ∼ 5000 theronts per fish, which were added directly into the aquarium. Then, tissue samples were taken at 0.5, 1, 4, 7, 14, 21, and 28 DPI (infected fish). Every experiment was performed at least three times. Control fish were maintained in a similar tank, but without parasites. The fish were starved on the days when sampling was conducted.

### Histology, light microscopy, and immunofluorescence microscopy studies

The OM tissue of rainbow trout was cut accurately, and the eyeball and muscle tissue beneath the mucosal layer removed carefully using anatomical scissors. Then, the tissues were processed for routine histology as described previously (Kong et al. 2019; Yu et al. 2018, 2019). Briefly, all OM tissue samples were fixed in 4% neutral buffered formalin (1:10) overnight at 4 °C and then were dehydrated through an alcohol gradient, mounted in paraffin, and cut into 5 μm slices. Sections were stained with hematoxylin–eosin (H&E, Biosharp, China) and alcian blue (A&B, Servicebio, China), which was used to check that most of the goblet cells of the OM were blue stained (acidophilic). Quantification was performed by three different researchers as in the previous study (Zhang et al. 2018). Immunofluorescence staining was employed to observe the location and distribution of Ich. The OM sections were incubated with anti-Ich polyclonal Ab (mouse IgG1, 1 μg ml^−1^) or isotype control mouse IgG1 overnight at 4 °C. Nuclei were stained with DAPI (Invitrogen, USA) before mounting. Images were acquired and analyzed using the Olympus BX53 fluorescence microscope and the iVision-Mac scientific imaging processing software (Olympus).

### RNA isolation and qPCR analysis

Tissues were loaded into 1.5 mL RNase-free EP tubes containing 1 mL Trizol (Invitrogen) for RNA isolation according to the manufacturer’s instructions. The concentration and integrity of the RNA were examined by use of a NanoDropND-1000 spectrophotometer (Thermo Scientific) and gel electrophoresis. Equivalent amounts of each sample of RNA (1 μg) were used for cDNA synthesis following the Hifair III 1st Strand cDNA Synthesis Supermix (Yeasen, Shanghai) instruction book. Then, all of the synthesized cDNA was stored at − 20 °C. The qPCRs were performed on a 7500 Real-Time PCR System (Applied Biosystems) using 2 × SYBR Green qPCR Master Mix (Yeasen). All samples were examined as follows: 95 °C for 5 min, followed by 40 cycles at 95 °C for 10 s and 58 °C for 30 s. Relative mRNA abundance was calculated using the 2^−ΔΔ*Ct*^ method and normalized to EF1α, whereas the relative abundance of Ich was shown as − ΔΔ*Ct* (Livak and Schmittgen 2001). Details about the primers used for qPCR are provided in Supplementary Table S1.

### RNA-Seq library construction, sequencing, and analysis

Samples from the control and Ich-infected groups were sent to Majorbio Bio-Pharm Technology Co. Ltd. (Shanghai, China) for RNA-Seq analysis; three replicates per sample. RNA-Seq libraries were prepared and analyzed as previously described. The prepared libraries were then sequenced using the Illumina HiSeq X Ten/NovaSeq 6000 sequencer to generate paired-end reads with a length of 150 bp. The raw reads were trimmed and quality controlled using SeqPrep and Sickle, and the remaining clean reads were mapped to the genome assembly of *Oncorhynchus mykiss*. Genes were considered as the differentially expressed genes (DEGs) if false discovery rate (FDR) ≤ 0.05 and |log2 (fold-change) |≥ 1.

### Statistical analysis

Unpaired Student’s *t* test (Prism version 8.0; GraphPad) was used to evaluate the differences between the OM sites or groups Data are expressed as mean ± SEM. All *P* values < 0.05 were considered statistically significant.

## Results

### Rainbow trout OM as the portal of Ich parasite infection

**Fig. 1:**
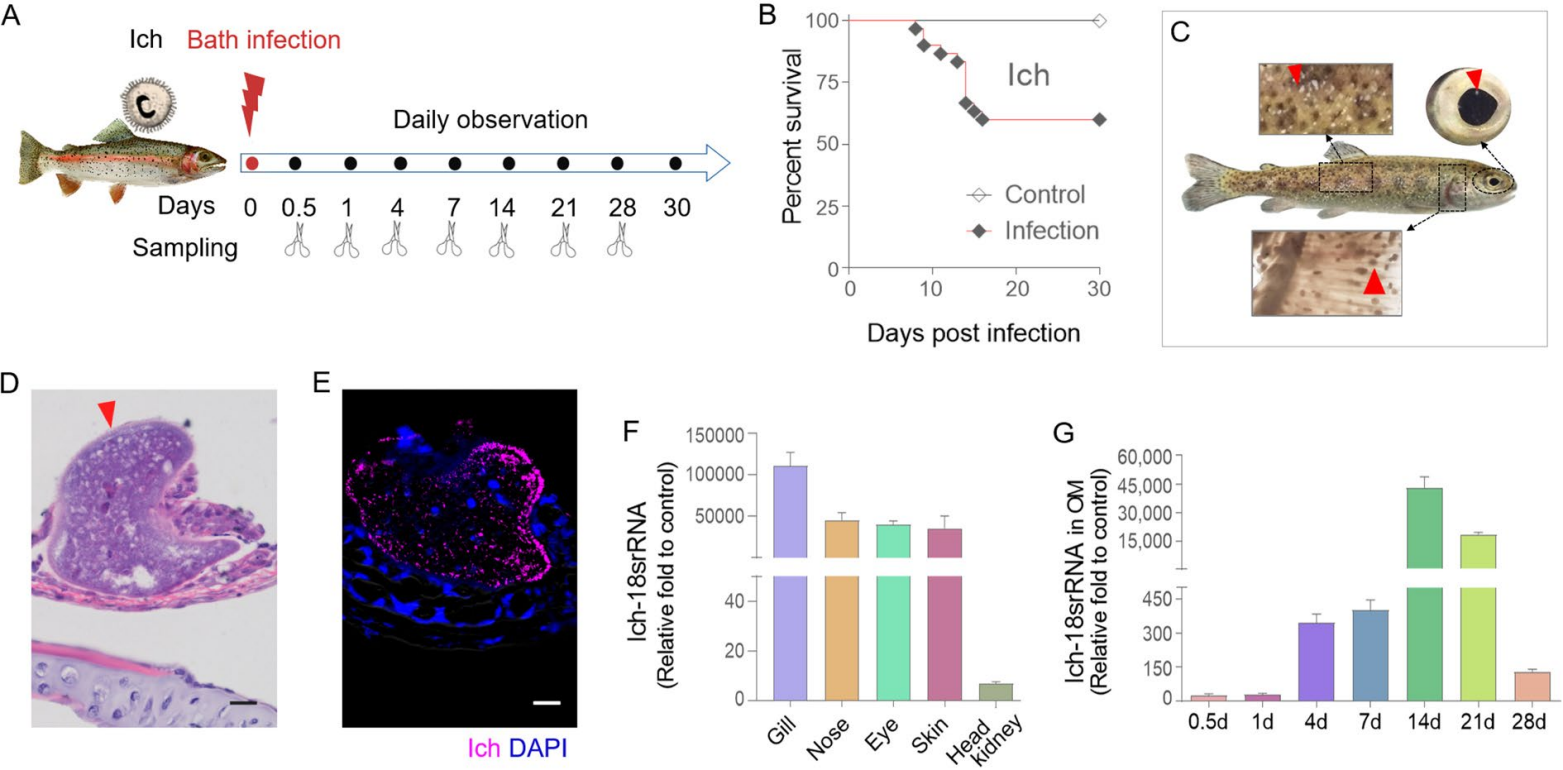
Establishment of Ich-infected rainbow trout model. **A** Rainbow trout was infected by Ich bathing for 4 h and then euthanized at 0.5, 1, 4, 7, 14, 21, and 28 DPI to collect tissue samples. **B** Cumulative survival of control and Ich-infected fish. **C** External morphological changes of rainbow trout infected with Ich. **D** H&E staining of OM of rainbow trout infected with Ich. **E** Immunofluorescence of OM of rainbow trout infected with Ich. Rainbow trout OM sections at 14 DPI were stained with anti-ich antibody (magenta) and the DNA-intercalating dye DAPI (blue). Scale bars, 20 µm. **F** The expression of Ich-18SrRNA in each organ was detected by qPCR at 14 DPI (*n* = 6 fish per group). **G** The expression of Ich-18SrRNA in trout OM was detected by qPCR (*n* = 6 fish per group)

**Fig. 2:**
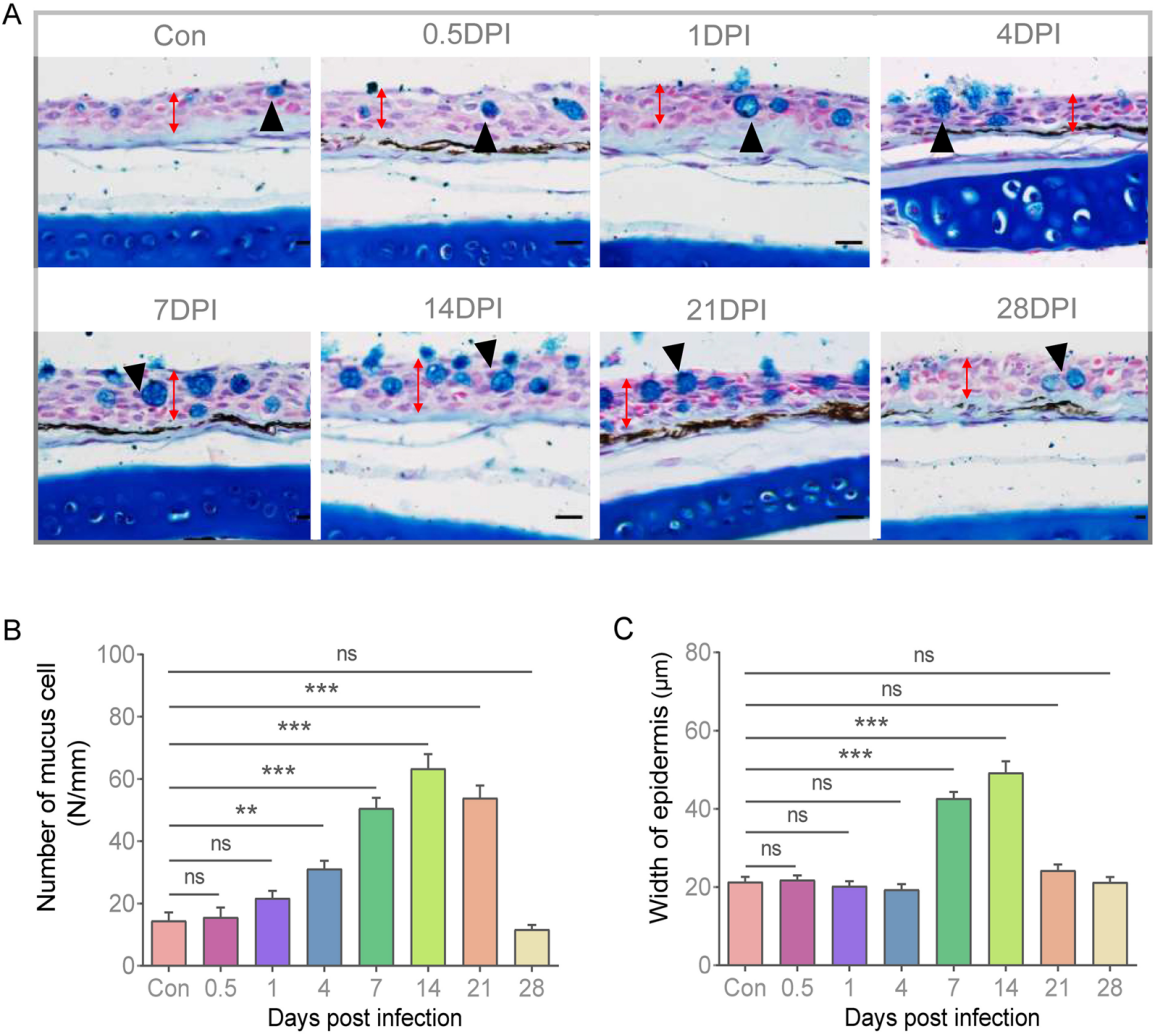
Pathological changes in OM of trout following Ich parasites infection. **A** Histological examination (alcian blue staining) of OM from trout infected with Ich at 0.5, 1, 4, 7, 21, 28 DPI and control fish (*n* = 6 fish per group). The red line with double arrows indicates the width of the OM epidermis. Black triangles indicate mucus cells. **B** The number of mucus cells per millimeter in OM tissue of control and infected fish (*n* = 6 fish per group). **C** The width of OM epidermis of control and infected fish (*n* = 6 fish per group). Scale bars, 50 µm. **P < 0.01, ***P < 0.001(unpaired Student’s *t*-test). Data are presented as mean ± SEM of three independent duplicates

To evaluate whether teleost OM elicits mucosal immune responses to parasite infection, we established an Ich immersion infection model in rainbow trout (Fig. 1A). The fish were exposed to an optimal single dose of ∼ 5000 theronts per fish, and then divided into two groups. The survival of one group of fish was monitored daily, whereas the other group of fish was sacrificed to collect tissue samples at 0.5, 1, 4, 7, 14, 21, and 28 days post-infection (DPI) (Fig. 1A). Over the 30 day infection period, the cumulative mortality rate of trout reached 42%, with the death rate stabilizing at 14 DPI (Fig. 1B). Clinical symptoms centred on the presence of small white spots that developed on the skin, gills and eye surface of infected fish at 14 DPI (Fig. 1C). Furthermore, using H&E staining and immunofluorescence, Ich was detectable within the OM epithelium (Fig. 1D, E). Moreover, we detected a higher expression of Ich-18SrRNA in the mucosal tissues (i.e., gills, nose, OM and skin) compared to the internal organs (i.e., head kidney) at 14 DPI (Fig. 1F). Importantly, the Ich load in OM was comparable with other mucosal tissues; one of the target organs of Ich, that indicated OM could be considered as an invasion site for parasites. To gain further insights into the histological changes of OM after Ich infection, sections of eye tissues were stained with A&B (Fig. 2A). Thus, the number of mucus cells in the OM exhibited a substantial increase at 14 DPI, with a return to normal levels by 28 DPI (Fig. 2B). Moreover, the epithelial tissue exhibited significant thickening at 14 DPI, after which it normalized by 21 and 28 DPI (Fig. 2C). These findings suggest that the OM represents a significant target for Ich invasion, and the tissue of the OM may hold a key role in resisting Ich infection.

### Immune responses in the trout OM upon Ich infection

**Fig. 3:**
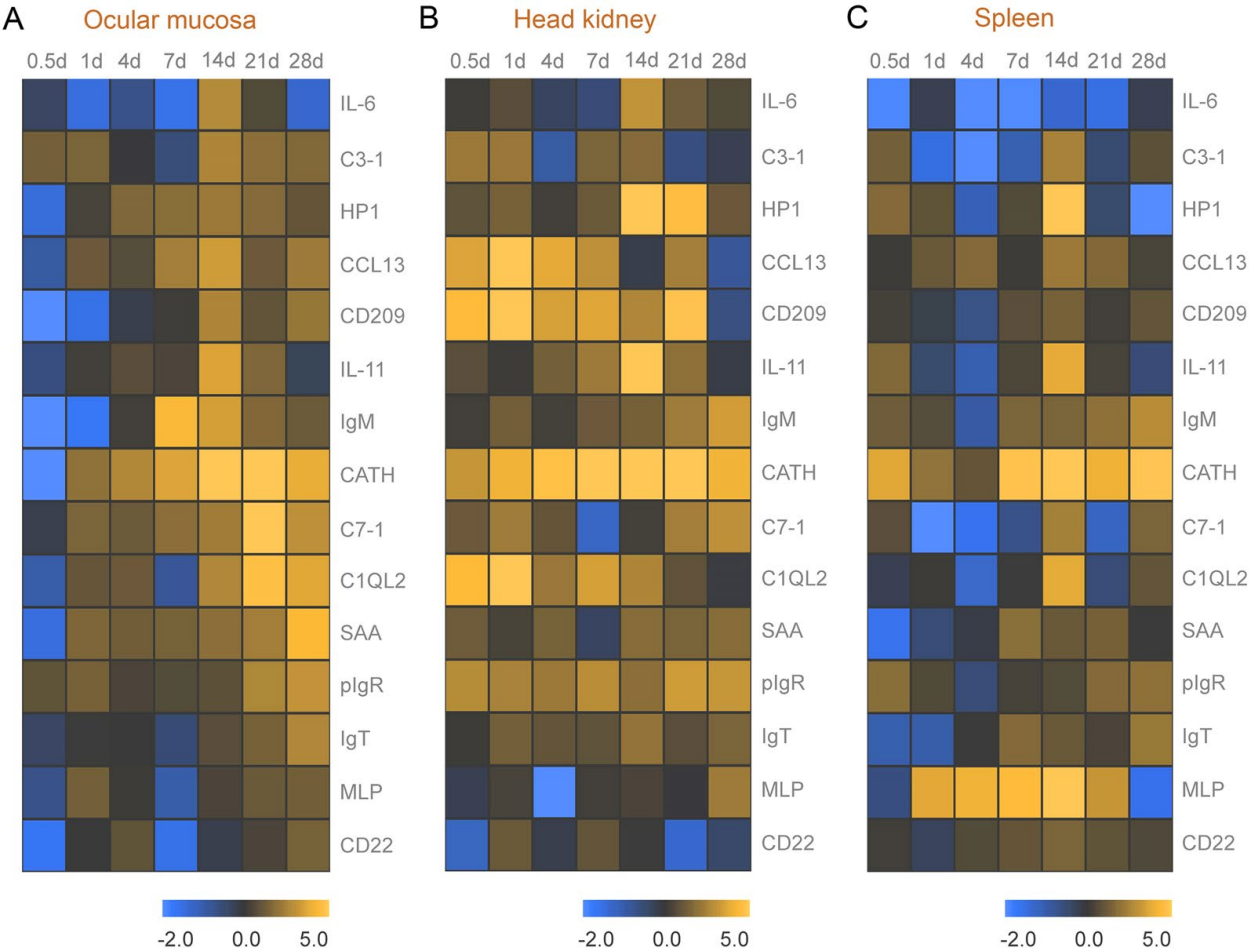
Kinetics of immune responses in OM of trout following Ich parasites infection. **A**–**C** Heatmap was used to picture heat maps to illustrate the kinetics of immune responses in trout ocular mucosa (**A**), head kidney (**B**), and spleen (**C**) of Ich-challenged vs control group measured at 0.5, 1, 4, 7, 21, and 28 DPI with Ich parasites (*n* = 6 fish per group)

To further investigate the kinetics of immune response in trout by qPCR, we analyzed the mRNA expression patterns of immune-related genes in trout OM during Ich infection. Remarkably, qPCR results revealed vigorous immune responses in the OM as well as other immune organs (head kidney and spleen). At 7 and 14 DPI, innate immune-related genes such as complement 3 (C3-1), C7-1, interleukin 6 (IL-6), haptoglobin 1 (HP1), C–C Motif Chemokine Ligand 13 (CCL13), IL-11, and Complement C1q Like 2 (C1QL2) exhibited high expression levels in the OM. Adaptive immune-related genes, such as Immunoglobulin T (IgT), poly immunoglobulin receptor (pIgR), Siglec 2 (CD22), serum amyloid A (SAA) and IgM, exhibited high levels at 28 DPI (Fig. 3A). Meanwhile, CD209 could be detected at 14 and 28 DPI, suggesting various adaptive/innate immune cells may participate in the immune response. Interestingly, IgM was also highly expressed at 7, 14, and 28 DPI, indicating that this immunoglobulin plays a role in both early and late parasitic infection. Otherwise, the immune genes in the head kidney and spleen were up-regulated after infection, but their gene expression patterns differed from those in the OM. Some immune-related genes were significantly increased in the head kidney at 0.5 or 1 DPI, which was earlier than that in trout OM and spleen (Fig. 3B, C). In addition, we found that the immune response occurring in the spleen was relatively weak (Fig. 3C). It is noteworthy that days 14 and 28 were the most relevant in terms of the intensity of the immune response in trout OM. Therefore these time points were selected for subsequent RNA-seq analysis.

### Analysis of transcriptomic changes in the OM after Ich infection

To further investigate the kinetics of immune response in trout OM, 12 samples from the two-time points mentioned above were divided into four groups (ICHC14d, ICHE14d, ICHC28d, and ICHE28d) for RNA-sequencing. A total of 367,531,421 high-quality clean data were obtained after a series of quality controls, and the percentage of Q30 base in all samples was not less than 90.77% (Supplementary Table S2). Further comparison of transcriptome data with reference genome sequences showed that the efficiency of comparison between Reads and reference genome of each sample ranged from 66.74 to 70.31%, and unique mapped reads ranged from 61.68 to 64.88% (Supplementary Table S3). Then, to assess the adequacy of the data and meet the requirements for subsequent analysis, the number of genes sequenced was tested for saturation. Thus, the results indicated that the amount of gene data with different expression levels could satisfy further analysis (Supplementary Fig. S1). The validated transcriptome libraries would be further analyzed for differentially expressed genes.

**Fig. 4:**
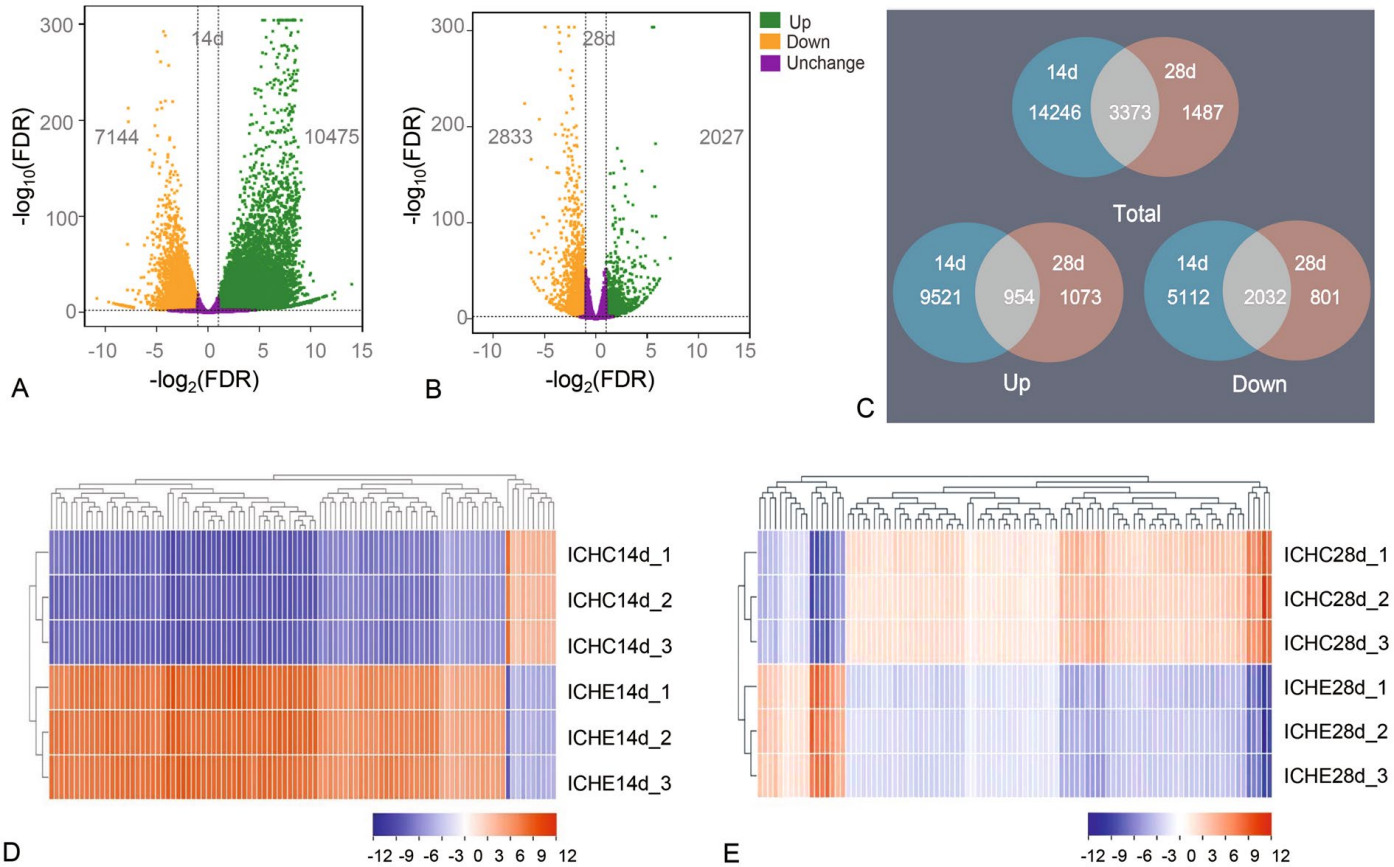
Effects of Ich-stimulation on the longitudinal transcriptomic dynamic changes of trout OM. **A**, **B** Volcano plot displaying the DEGs distribution in Ich-challenged group vs control group at 14 DPI (**A**) and 28 DPI (**B**). **C** Venn diagram of RNA-Seq experiments showing overlap of up- or down-regulated genes in rainbow trout OM at 14 or 28 DPI compared to control fish (*n* = 3 fish per group). Green spots, expression fold change of > 2 and FDR of < 0.05; Orange spots, expression fold change of < 2 and FDR of < 0.05; Purple spots, no difference in expression. Up, up-regulated differential genes; Down, down-regulated differential genes. **D**, **E** Heat map analysis of the top 100 DEGs in Ich-challenged group vs control group at 14 (**D**) and 28 DPI (**E**), respectively

Differential expression analysis was conducted using DESeq2 to compare gene expression patterns between different sample groups, aimed to identify genes that exhibited significant differences between two distinct biological conditions. In the process of differential expression gene detection, Fold Change ≥ 2 and FDR < 0.01 were used as screening criteria. Transcriptome analyses revealed a total of 17,619 DEGs in the ICHE14d/ICHC14d group (10,475 up-regulated and 7144 down-regulated), and 4860 DEGs in the ICHE28d/ICHC28d group (2027 up-regulated and 2833 down-regulated) (Fig. 4A, B). Between ICHE14d/ICHC14d group and the ICHE28 d/ICHC28d group, an overlap of 3373 DEGs was observed (954 up-regulated and 2,032 down-regulated) (Fig. 4C). Then, we analyzed and compared the top 100 DEGs at 14 and 28 DPI, finding that the quantity and intensity of DEGs was greater at 14 DPI compared to 28 DPI (Fig. 4D, E). To validate the RNA-Seq expression levels, 11 genes were selected to validate by using qPCR. Linear regression analysis conducted between the transcriptome results and qPCR data demonstrated a strong consistency (R = 0.95) confirming the reliability of the transcriptome data (Supplementary Fig. S2).

### Functional enrichment analysis of differentially expressed genes

**Fig. 5:**
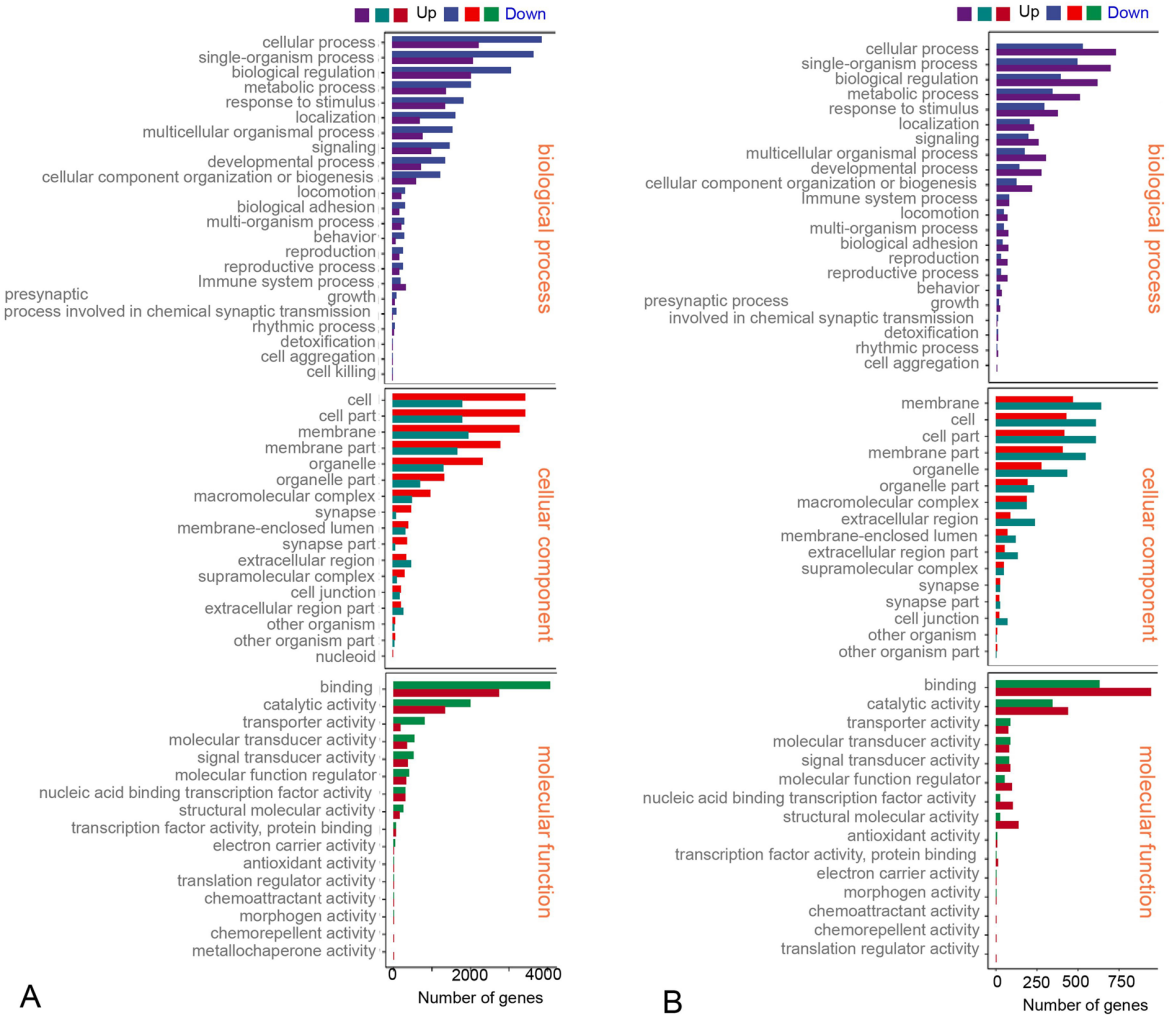
GO enrichment analysis of Ich-stimulation on the transcriptomic changes in the OM of rainbow trout. **A** Ich-challenged group vs control group at 14 DPI. **B** Ich-challenged group vs control group at 28 DPI. The y-axis represents the Gene Ontology process, and the x-axis represents the number of genes in the process

Subsequently, we annotated the screened differential genes using different databases. Thus, 17,788 DEGs were annotated, and the results of each annotation are shown in Supplementary Table S4. A total of 13,647 DEGs had GO annotation, and were classified into 56 functional items (Supplementary Table S4). Ontology (GO) enrichment analysis revealed the top three enriched pathways within the “biological process,” “cellular component,” and “molecular function” categories (biological process: cellular process, single-organism process, and biological regulation; cellular component: cell, cell part, and membrane; molecular function: binding, catalytic activity, and transporter activity) (Fig. 5A, B).

**Fig. 6:**
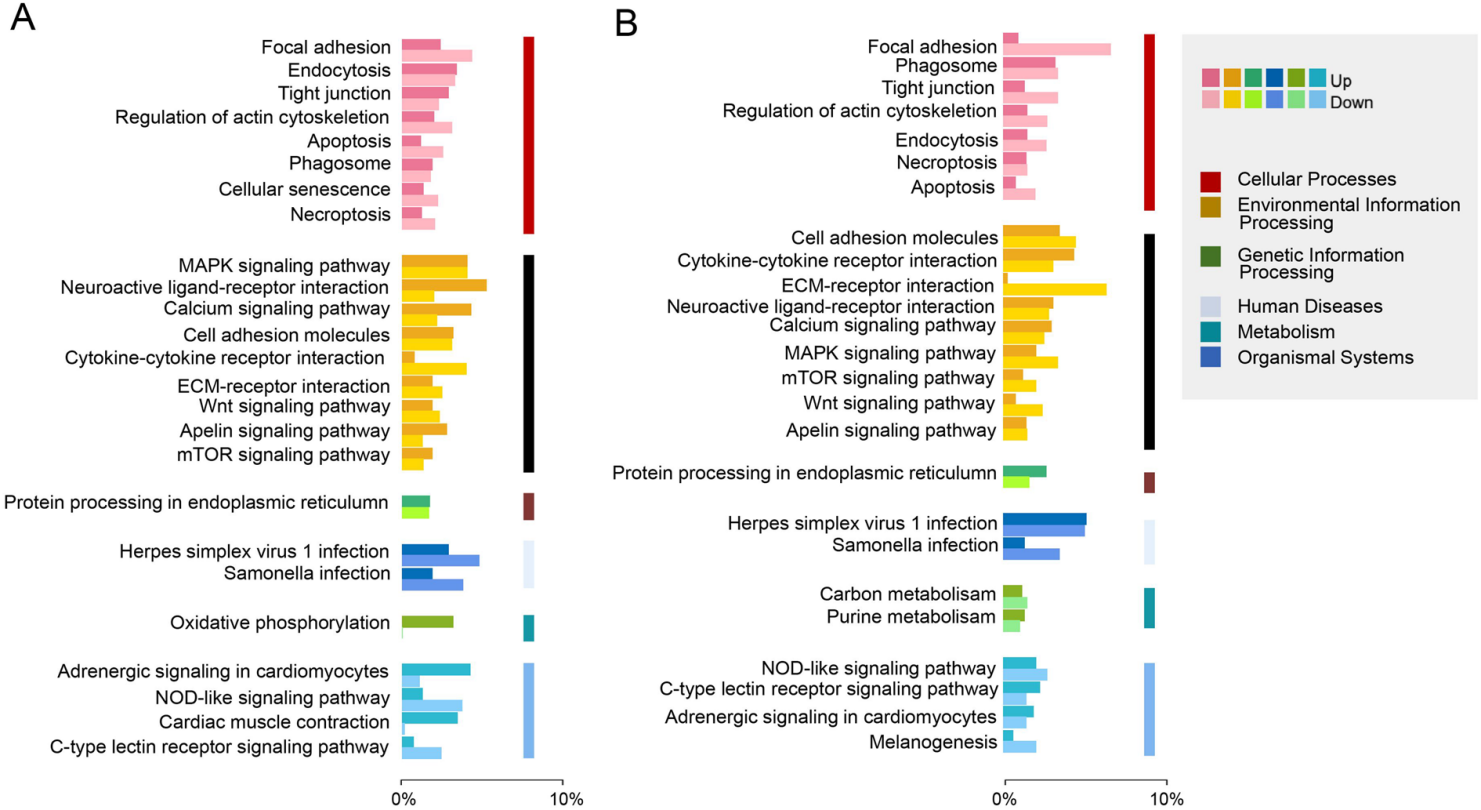
KEGG enrichment analysis of Ich-stimulation on the transcriptomic changes in the OM of trout. **A** Ich-challenged group vs control group at 14 DPI. **B** Ich-challenged group vs control group at 28 DPI. Genes are assigned to six special KEGG pathways, including cellular processes, environmental information processing, genetic information processing, human disease, metabolism, and organismal systems

Additionally, DEGs were subjected to a search in the Kyoto Encyclopedia of Genes and Genomes (KEGG) database for functional prediction and classification (Fig. 6A, B). A total of 14,644 DEGs had KEGG annotation and were classified into 25 functional items (Supplementary Table S4). The KEGG analyses revealed that the DEGs were predominantly associated with cellular processes and environmental information processing. Furthermore, compared to the 28 DPI, the DEGs at 14 DPI exhibited more pronounced enrichment responses in pathways including focal adhesion, MAPK (Mitogen-Activated Protein Kinase), mTOR (Mammalian Target of Rapamycin), calcium signaling, and the C-type lectin receptor signaling pathway.

### Analysis of immune-related pathways in the transcriptome

**Fig. 7:**
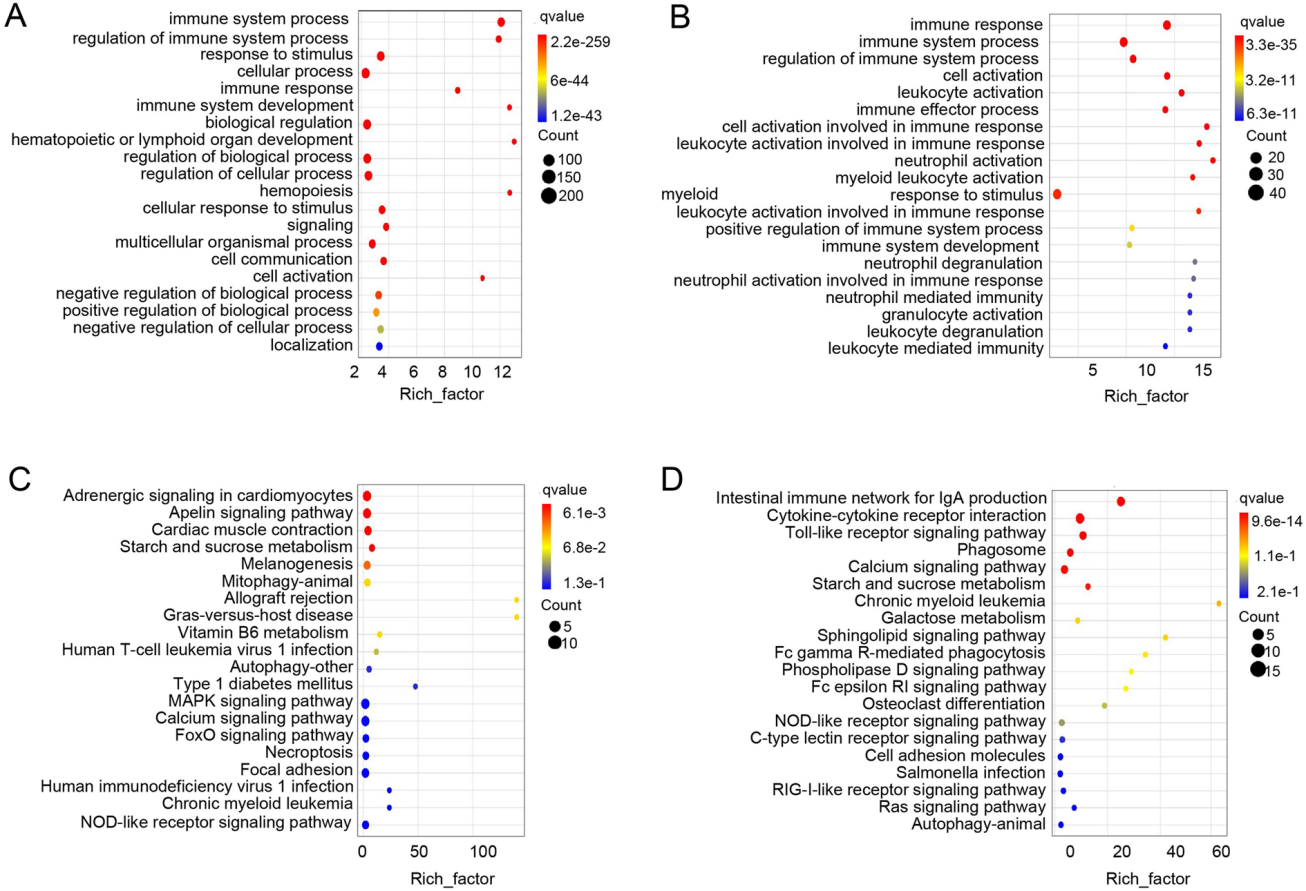
Immune-related pathway enrichment analysis in the OM of trout after Ich infection. **A**, **B** GO enrichment analysis of up-regulated immune-related genes in the OM of trout at 14 DPI (**A**)and 28 DPI (**B**). **C**, **D** KEGG enrichment analysis of up-regulated immune-related genes in the OM of trout on 14 DPI (**C**) and 28 DPI (**D**)

**Fig. 8:**
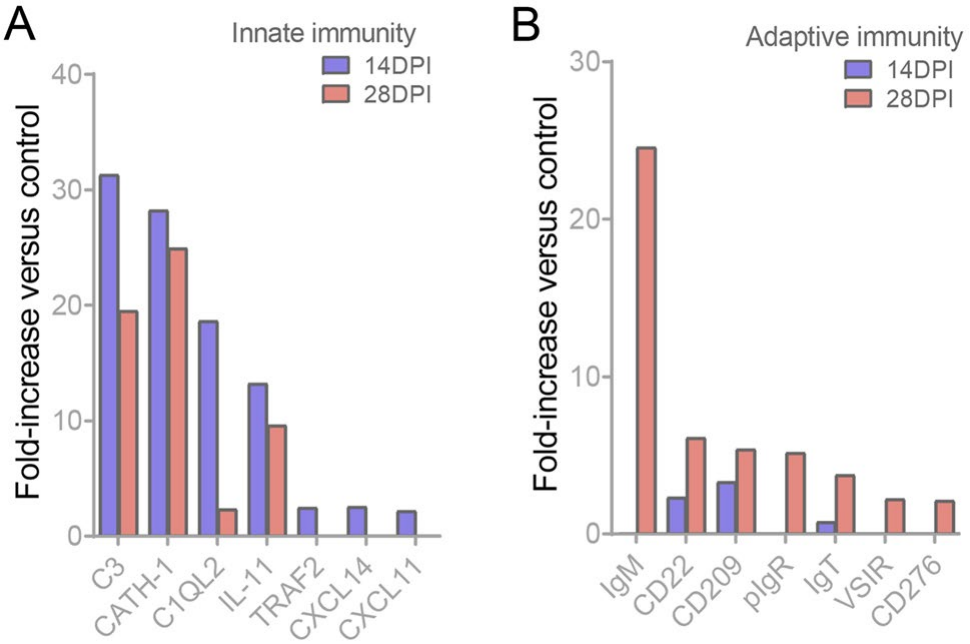
The changes of innate and adaptive immune-related genes in OM of trout after Ich infection. **A**, **B** Expression fold changes of innate (**A**) and adaptive (**B**) immune marker genes at 14 and 28 DPI

A comprehensive transcriptional analysis was conducted to gain insights into the intricacies of ocular immune-related processes. Up-regulated expression immune-related genes identified in the 14 and 28 DPI groups were further analyzed using the GO and KEGG databases. These analyses revealed significant variations in immune processes between the two-time points. At 14 DPI, the GO analysis highlighted an abundance of genes enriched in cellular processes, cellular responses to stimuli, and the regulation of cellular processes (Fig. 7A). This observation may be linked to the proliferation and differentiation of mucosal cells, including the increased presence of mucous cells after infection. The KEGG results revealed that the top three most affected pathways included the apelin signaling pathway, starch and sucrose metabolism, and adrenergic signaling in cardiomyocytes (Fig. 7C). It is likely that these pathways provide the foundational elements for mucosal cell proliferation, differentiation, and reparative processes. Notably, certain pathways associated with negative regulation of biological and cellular processes were also found to be enriched. At 28 DPI, GO analysis revealed changes primarily in biological process gene pathways associated with the immune response, immune system process, and cell and leukocyte activation, all of which are involved in the immune response (Fig. 7B). According to KEGG enrichment analysis, the intestinal immune network for IgA production, cytokine-cytokine receptor interaction, and the toll-like receptor signaling pathway were identified as prominent players in the immune response (Fig. 7D). Further analysis of key molecules within the context of innate and adaptive immunity at 14 and 28 DPI yielded important insights (Fig. 8A, B). Notably, at 14 DPI, C3, CATH-1, and C1QL2 were upregulated, whereas at 28 DPI, IgM, CD22, CD209, pIgR, and IgT exhibited increases in relative expression. Conversely, TRAF2, CXCL14, and CXCL11 demonstrated lower expression across both time points. These findings collectively suggest that during the initial stages of infection, Ich is primarily eliminated through innate immune mechanisms, such as complement molecules, antimicrobial peptides and lectins, whereas acquired immunity components, such as IgM and IgT, exert a more significant role in the later stages of infection.

### Adverse effects of Ich infection on the eye of rainbow trout

**Fig. 9:**
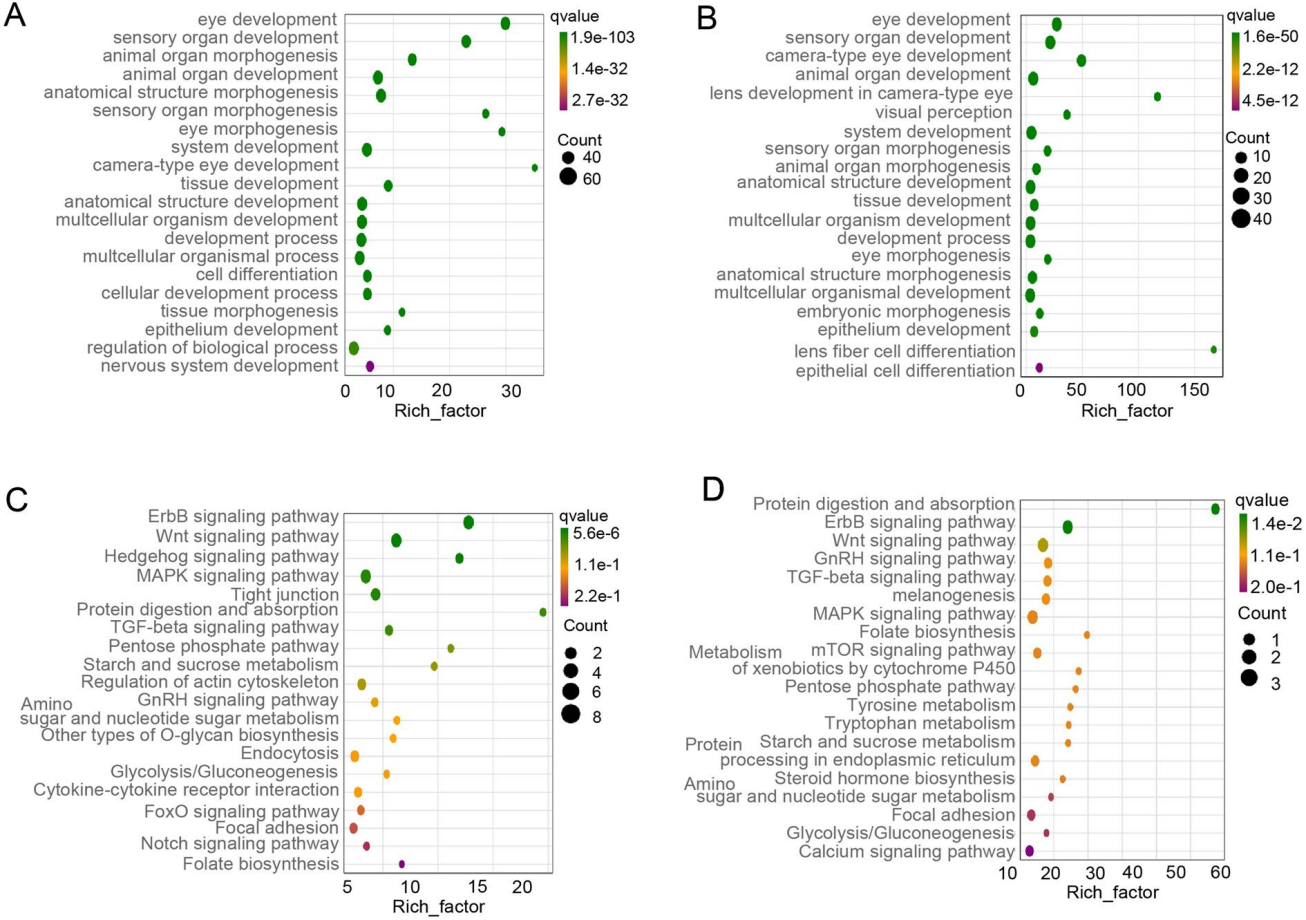
Biological function enrichment analysis in the OM of trout after Ich infection. **A**, **B** GO enrichment analysis of down-regulated eye function-related genes in the OM of trout at 14 DPI (**A**) and 28 DPI (**B**). **C**, **D** KEGG enrichment analysis of down-regulated eye function-related genes in the OM of trout on 14 DPI (**C**) and 28 DPI (**D**)

**Fig. 10:**
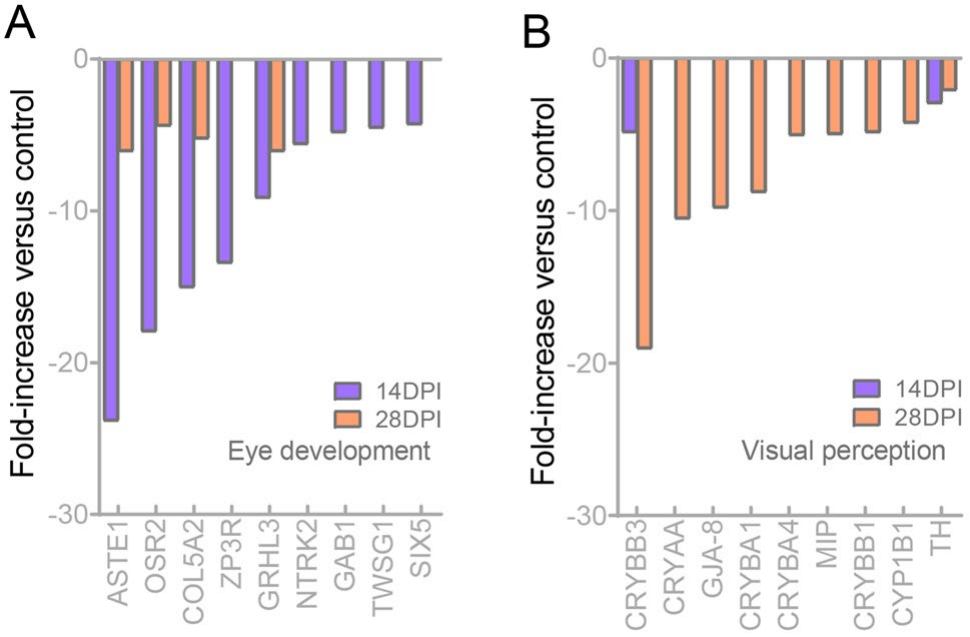
The changes of visual function-related genes in the OM of trout after Ich infection. **A**, **B** Expression fold changes of eye development (**A**) and visual perception (**B**) related genes at 14 and 28 DPI

To determine whether the eye function is affected during the immune process of the OM against Ich, we further analyzed the relevant differential genes. Therefore, our study analyzed the down-regulated expression of genes associated with eye development and visual perception within the transcriptome. The results of the GO analysis revealed response processes including eye development, sensory organ development and animal organ morphogenesis (Fig. 9A, B). Notably, DEGs in the 14 DPI exhibited more pronounced changes. KEGG analysis identified the ErbB, Wnt and MAPK signaling pathways as potential key players in the restoration of eye function (Fig. 9C, D). Additionally, we examined the changes in the regulation of key molecules in response to Ich infection (Fig. 10A, B). At 14 DPI, ASTE1, OSR2, COL5A2, ZP3R, GRHL3, NTRK2, GAB1, TWSG1 and SIX5 displayed more significant down-regulation. Conversely, CRYBB3, CRYAA, GJA-8 and MIP showed sharper decreases. Taken together, our findings demonstrated that the eye endured more severe and extensive damage at 14 DPI compared to 28 DPI, with a greater response observed among genes related to the functional and structural aspects of eye tissue.

## Discussion

The OM of vertebrates is an important entry point for various pathogens, such as viruses, bacteria and fungi (Armstrong et al. 2021; Klotz et al. 2000; KrishnanNair and Solai 2022), which produces innate and adaptive immunity, thereby protecting eye function (Knop and Knop 2000; van Ginkel et al. 2012). So far, local mucosal responses in the OM surface have only been described in tetrapods. However, the immune function of the OM in the early vertebrates like teleost fish against pathogens, especially parasites, is not well understood. In this study we are the first to provide evidence that the OM is susceptible to parasitic infections, prompting robust local immune responses.

To evaluate whether the trout OM expresses immune-related genes after parasite infection, we established an Ich parasite infection model in rainbow trout, via immersion, as evidenced by the results of H&E staining and immunofluorescence analyses. Earlier research identified gills, nose and skin mucosal tissue as primary sites for Ich invasion (Xu et al. 2016; Zhang et al. 2018). Accordingly, we observed that the body surfaces of infected fish exhibited the typical clinical symptoms of many small white spots, including eyes, skin and gills. Notably, the Ich load in the OM of surviving fish began to increase significantly at 4 DPI and peaked at 14 DPI similar to previously reported in the nose and pharynx of trout (Kong et al. 2019; Yu et al. 2018). Additionally, A&B staining revealed an increase in mucous cell numbers and thickening of the OM. These results suggest that the OM may be one of the primary targets of the parasites, resulting in pathological damage to the eye. By qPCR, we revealed alterations in immune-related genes within the OM, head kidney and spleen, thus confirming the activation of local immune responses by Ich. Moreover, we found that a large number of genes, such as IgT and pIgR, associated with innate and adaptive immunity were significantly elevated at 14 and 28 DPI, respectively. This is in agreement with previously reported findings in buccal mucosa and pharynx (Kong et al. 2019; Yu et al. 2019). Previous studies have shown that, like mammalian IgA, sIgT is the main player involved in parasite-specific mucosal immune responses (Kong et al. 2019; Xu et al. 2013, 2016; Yu et al. 2018, 2019). It is noteworthy that IgM, an adaptive immune molecule, is responsive to Ich parasites in trout OM at 7 DPI. Future studies will explore the specific role of IgM against parasitic infections at the early stage. These findings suggest the existence of immune processes within the OM likely playing a pivotal role in maintaining ocular homeostasis.

Next, we used the transcriptome to characterize the overall gene expression in Ich-infected rainbow trout at 14 and 28 DPI. The transcriptome sequencing analysis highlighted the occurrence of a general view of the biological processes in trout OM upon Ich infection. Accordingly, we identified 17,619 DEGs in the ICHE14d/ICHC14d and 4860 DEGs in the ICHE28d/ICHC28d group following Ich infection. The difference is that the number of DEGs in the trout OM was much higher than in buccal and pharyngeal cavities at the same time points (Kong et al. 2019; Yu et al. 2019), indicating significant differences in the degree of mucosal response to parasitic infection at different anatomical locations. Moreover, GO enrichment analysis demonstrated that biological processes representing genes were involved in cellular processes, response to stimulus, developmental processes, single-organism processes, immune system processes, etc. Most notably, the number of genes associated with the immune system process and response to stimulus in trout OM at the 14 DPI was significantly higher than that at 28 DPI. This result suggested trout OM mounted a more pronounced and stronger immune response to Ich parasite infection at 14 DPI than that at the 28 DPI aligning with the results we obtained by qPCR data and Ich load. This result also means that the immune response was already in the “adaptive phase” at 28 DPI. Importantly, KEGG enrichment analysis showed that two types of programmed cell death pathways (i.e., apoptosis, and necroptosis) were activated in trout OM after Ich infection, all of which were involved in the cell turnover and repairing the tissue damage at a late stage (Cordero et al. 2017; Easy and Ross 2009; Fernández-Montero et al. 2021; Provan et al. 2013). Collectively, our findings suggest that Ich infection could induce a strong mucosal immune response and might damage trout’s eye tissue and even affect its visual function. In subsequent analyses, we focused on the expression of genes associated with immune response processes upon Ich parasite infection, as well as the signal responses tied to eye injury.

To explore the differences between early and late mucosal immune responses, we analyzed immune-related biological processes and signaling pathways following Ich infection. At 14 DPI, GO enrichment analysis indicated that biological processes representing genes were involved in the regulation of immune system processes, response to stimulus, cellular processes and immune response. KEGG analysis highlighted the Adrenergic signaling in cardiomyocytes, which may be related to the regulation of anti-inflammatory response. In rodents, this response through adrenaline-related signaling pathways also plays an important role in regulating inflammation in chronic toxoplasma infection (Laing et al. 2020). Interestingly, glucose, lipid and amino acids metabolism-related pathways (i.e., Apelin signaling pathway, Cardiac muscle contraction, Starch and sucrose metabolism and vitamin B6 metabolism) were also annotated. However, during *Plasmodium* infection, hypometabolic responses temporarily inhibit hepatic glucose production to prevent unconstrained immune-mediated inflammation, organ damage, and anemia (Ramos et al. 2022). Also, it is noteworthy that pathways negatively regulating cellular and biological processes were also enriched, which may be linked to the suppression of ocular inflammation, as uncontrolled ocular inflammation can lead to blindness (de Paiva et al. 2022). To further understand the regulation of inflammation in the immune process in the OM, we analyzed the important immune molecules involved such as C3, CATH-1, C1QL2, IL11, TRAF2, CXC14, and CXC11. C3, CATH-1 and C1QL2. We found that Ich invasion of OM induces low-level inflammation, which may be related to the maintenance of inflammatory homeostasis by the anti-inflammatory factor IL11 (Ip et al. 2017). Crucially, C3 had a high expression level at 14 DPI. In our previous report, the complement system was also significantly upregulated in the skin mucosa invaded by Ich, which may play a vital role in both innate and adaptive immunity (Nakao et al. 2011; Zhang et al. 2018). Next, we analyzed immune-related biological processes and signaling pathways following Ich infection at 28 DPI. GO analysis revealed that the top 4 are immune response, immune system process, regulation of immune system process and cell activation. Unlike the analysis at 14 DPI, several lymphocyte-related signaling pathways are activated at 28 DPI. Signaling pathways related to IgA are activated, with IgA being a crucial antimicrobial element in mammalian tears (Brandtzaeg et al. 2001). In teleost fish, similar to mammalian IgA, the specific IgT is an indispensable part of mucosal immunity (Zhang et al. 2010, 2021). Moreover, IgM and pIgR were also significantly up-regulated, and they play important functions in the OM (Tongsri et al. 2020; Xu et al. 2021). These results suggest that lymphocytes and immunoglobulins may exert a crucial part in the late stages of infection. Meanwhile, a series of pattern recognition receptors are activated to the relevant pathways, for example, Toll-like receptor signaling pathway, NOD-like receptor signaling pathway, Fc gamma R-mediated phagocytosis, C-type lectin receptor signaling pathway and RIG-I-like receptor signaling pathway. These signaling pathways may mediate lymphocyte involvement in parasite clearance (William 2015). Certainly, these results suggest that acquired immunity is essential to the ocular mucosa. However, we cannot determine whether these lymphocytes migrate from other tissues or are native to the ocular mucosa, and these need to be further studied.

Parasites and cytokines are closely linked to pathological processes, and excessive immune responses may lead to adverse effects on eye function (MacDonald et al. 2002). GO analysis of DEGs related to ocular function showed that many down-regulated DEGs were enriched in the pathways related to ocular development and visual function after Ich infection. Notably, the downregulation of genes linked to eye development was more pronounced at 14 DPI than 28 DPI, in line with Ich quantities and immune response intensity at an earlier time point. By KEGG analysis at 14 DPI, the results indicated a lot of down-regulated DEGs were involved in the ErbB signaling pathway, Wnt signaling pathway, Hedgehog signaling pathway and MAPK signaling pathway. These pathways have a cross-effect between eye development and inflammatory response, such as the ErbB signaling pathway (Schumacher et al. 2017; Wieduwilt and Moasser 2008). We hypothesize that functional repair of the eye is hindered by the combination of parasite influence and inflammation suppression, but more evidence is needed to support our concerns. Moreover, an intriguing enrichment in gene pathways associated with nervous system development was observed at 14 DPI. At 28 DPI, GO analysis revealed that more important visual function pathways are down-regulated (i.e., camera-type eye development, lens development in camera-type eye, visual perception). Compared to 14 DPI, fewer down-regulated genes were annotated at 28 DPI. This indicates that the function of the OM is gradually recovering, which is consistent with the pathological changes. Similarly, the KEGG results revealed a large number of genes involved in the ErbB signaling pathway, Wnt signaling pathway, GnRH signaling pathway and TGF-beta signaling pathway. Importantly, protein digestion and absorption-related genes were down-regulated. Indeed, the down-regulation of amino acid uptake-related pathways may affect the proliferation, differentiation and repair of ocular mucosal cells (Ren et al. 2017; Sinclair et al. 2013). Otherwise, KEGG analysis results indicated the down-regulation of genes associated with melanogenesis, a process crucial for late-stage OM repair and restoration of visual function (D'Mello et al. 2016). Thus, our analysis reveals that when parasites compete with the ocular mucosal immune response, inflammation inhibition may affect the ocular mucosal repairing.

In conclusion, our findings provide the first evidence that the ocular mucosa can act as an important site of Ich invasion, which activates ocular mucosal immunity. The combined insight from qPCR and transcriptome data underscores the importance of both innate and adaptive immunity during the early and late phases of infection, hinting at the existence of EALT in bony fish. Furthermore, our results showed that the complement system and cathelicidin within the OM assume pivotal roles in countering parasitic infections. Notably, we found that some lymphocytes-associated molecular markers (e.g., IgT and IgM) were activated after parasite infection, indicating the involvement of B cells in teleost OM immunity. Therefore, future studies should focus on characterizing the role of IgM^+^/IgT^+^ immune cells in promoting ocular resistance against foreign invasive infections. Taken together, our finding provides novel insights into the cellular and molecular mechanisms of ocular mucosal immunity in teleost fish.

### Supplementary Information

Below is the link to the electronic supplementary material.

Supplementary file1 (DOC 1192 KB)

## Data Availability

All original data can be requested from the first author.
