# Supplementary material for: Mucosal immune responses to Ichthyophthirius multifiliis in the ocular mucosa of rainbow trout (Oncorhynchus mykiss, Walbaum), an ancient teleost fish
Source: Mar Life Sci Technol. 2023 Oct 31;6(2):266–79. doi: 10.1007/s42995-023-00199-6 (PMC11136906; doi:10.1007/s42995-023-00199-6)
Supplement: Supplementary file 1 — Supplementary file1 (DOC 1192 KB) [file 42995_2023_199_MOESM1_ESM.doc]

**Supplemental materials**

Transcriptomic analysis reveals innate and adaptive immune responses to *Ichthyophthirius multifiliis* in the ocular mucosa of rainbow trout (*Oncorhynchus mykiss*, Walbaum), an ancient teleost fish

Weiguang Kong^1^, Guangyi Ding^1^, Gaofeng Cheng^2^, Peng Yang^1^ and Zhen Xu^1*^

^1^Key Laboratory of Breeding Biotechnology and Sustainable Aquaculture, Institute of Hydrobiology, Chinese Academy of Sciences, Wuhan 430072, China

^2^Department of Aquatic Animal Medicine, College of Fisheries, Huazhong Agricultural University, Wuhan 430070, China

*Correspondence: Zhen Xu (zhenxu@ihb.ac.cn)

**
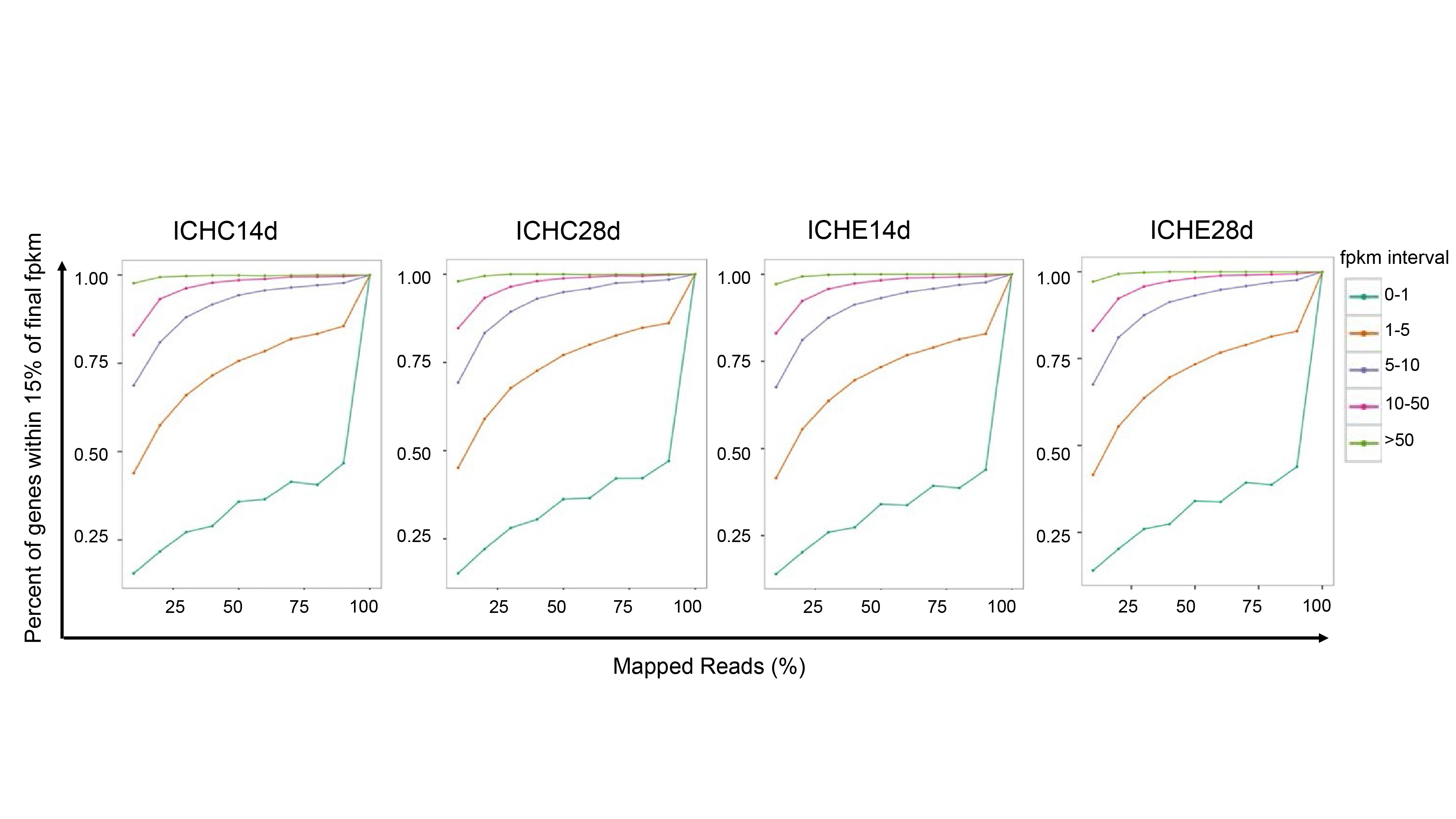
**

**Supplementary Fig. S1** Transcription group data saturation analog. Note: Random selection of 10%, 20%, 30%...... 90% of the total sequencing data were quantitatively analyzed separately. The horizontal coordinate represents the percentage of Reads located on the genome from the extracted data in the total number of reads located, and the vertical coordinate represents the percentage of Genes whose expression difference is less than 15% among all the sampled results in each FPKM range. The closer the value is to 1, the more saturated the expression and each color line represents the saturation curve of gene expression at different expression levels in the sample.


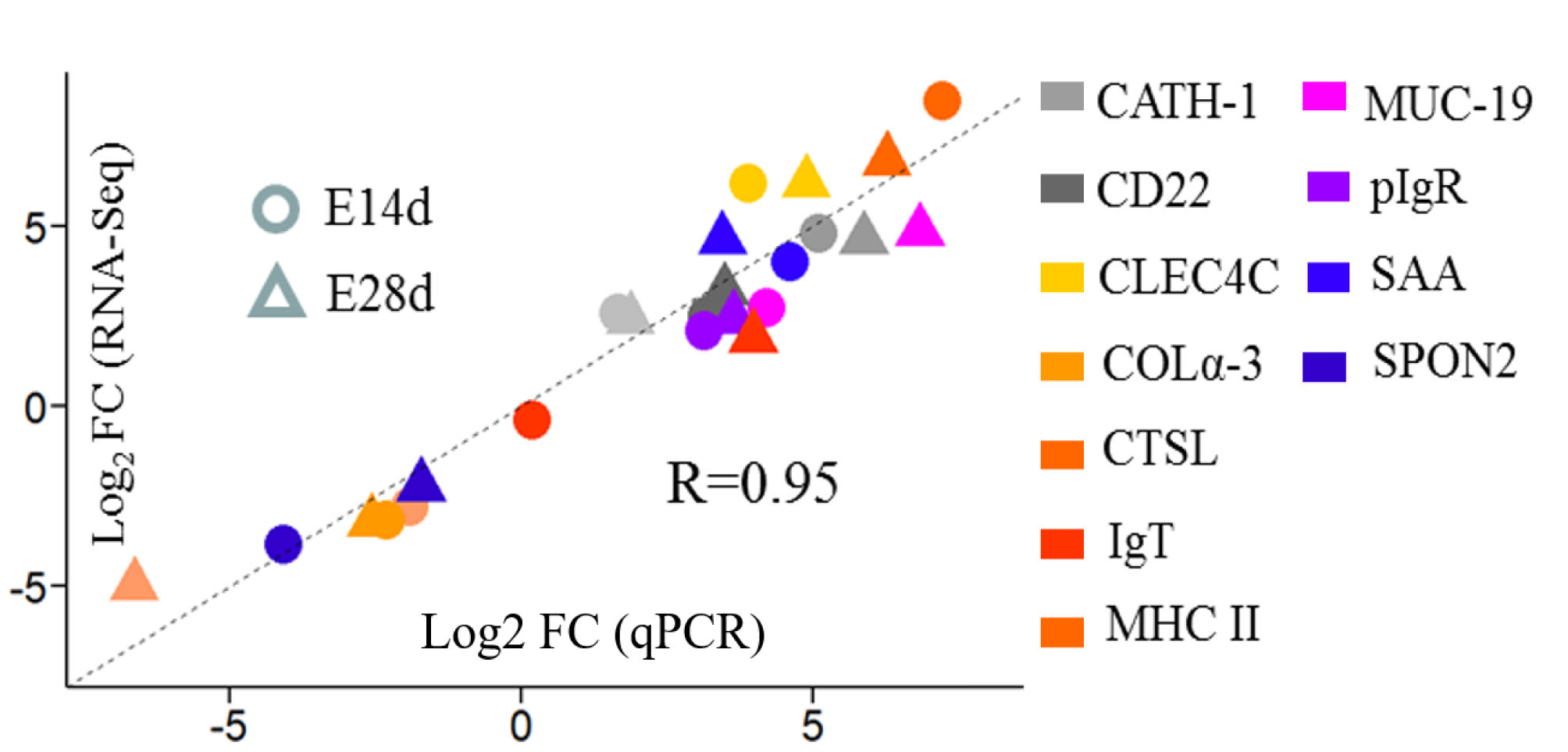


**Supplementary Fig. S2** Consistency analysis of transcriptome and RT-qPCR results.

**Supplementary Table S1** Primers used in this study.

| Gene | Primer Sequence (5’-3’) | |
| --- | --- | --- |
|  | Forward primer | Reverse primer |
| EF1α | GGCAAGCCCATGTGTGTGGA | ACCACCCGAGGGACATCCTG |
| IL-6 | GCTCGGGACAGACGGTGAAA | GAAACTCCTCCACAAACTGTTG |
| CATH | GAACACTGACCTGTCTACATTTG | GACACAATTTTTGCCTCTGGA |
| C3-1 | CCTCACAACAAGAGTGCACATC | CCAAGTGGGCAAACTCATCTCC |
| IgT | CAGACAACAGCACCTCACCTA | GAGTCAATAAGAAGACACAACGA |
| IgM | AAGAAAGCCTACAAGAGGGAGA | CGTCAACAAGCCAAGCCACTA |
| pIgR | GTACAGCAGGTGTTCACAGTAAC | CCACAGACGACCTTGGATAAC |
| C7-1 | TATCTTCACTGCCACGGTC | TAGCCTGTAACTCCACATAGAC |
| C1QL2 | TGAACAACCTGAACACCCC | CAGCCTATTAGCCTGTAACTCC |
| IL-11 | CAGAGCGTCAAGGAAACAC | GCTCCTGGGAAGACTGTAA |
| CD209 | GCCCTCTTTGTGCTGATT | ACTCCCTACACTTCCTTA |
| SAA | TTGTTCTGACCCTCGTTG | CCTGGCAGCATCATAGTT |
| MLP | GCTCTACTATCCAGCCAAC | GCATCCACAGTCACGAAC |
| CD22 | TGAAGATGACAGTGGCAGAT | GGAGGGTTACAGGTGGAG |
| CCL13 | CAGAACAACCTCCAGTAGC | ATCGTCGTCTTGGCAGTA |
| CATH-1 | CTGGAGGCAAGCAACAAC | CCCCCAAGACGAGAGACA |
| MUC-19 | TGGGTTTCTGTACGTCCGTG | GTGGCCCTGCTCATTGTAGT |
| THBS2 | TCCAGAACAACGAGGGCTTC | TGCGAGTCAGACAGGTCAAC |
| MHC Ⅱ | AATGGCGACTGGCACTA | GCCCGATGGCTATCTTA |
| CLEC4C | GCAGCCACCTTACCATC | CACCCATCTCCAATCCC |
| COLα-3 | TGCATTCCTGATTGACGGCT | AGAAATTGGGAGCTGGCCTC |
| CTSL | CTACGTGCCAGTGCTCAGAA | TGGAGCAGTCCACAAGGTTC |
| 18S rRNA | AGTGACAAGAAATAGCAAGCCAGGAG | ACCCAGCTAAATAGGCAGAAGTTCAA |

**Supplementary Table S2** Statistical output of sequencing data.

| Samples | Clean reads | Clean bases | GC Content | %≥Q30 |
| --- | --- | --- | --- | --- |
| ICHC14d1 | 28,478,079 | 7,891,464,065 | 48.25% | 94.75% |
| ICHC14d2 | 29,063,420 | 8,017,902,592 | 48.36% | 94.74% |
| ICHC14d3 | 29,684,195 | 8,230,230,637 | 48.11% | 94.81% |
| ICHC28d1 | 32,179,809 | 8,865,264,949 | 48.03% | 94.74% |
| ICHC28d2 | 28,974,766 | 8,009,837,451 | 48.11% | 94.69% |
| ICHC28d3 | 29,463,794 | 8,176,794,438 | 48.11% | 94.82% |
| ICHE14d1 | 30,634,793 | 8,520,309,470 | 47.52% | 94.84% |
| ICHE14d2 | 33,206,234 | 9,221,625,051 | 47.96% | 94.79% |
| ICHE14d3 | 28,775,351 | 7,987,385,954 | 47.90% | 94.74% |
| ICHE28d1 | 30,955,537 | 8,531,770,695 | 47.72% | 94.68% |
| ICHE28d2 | 33,830,106 | 9,317,732,304 | 47.69% | 94.76% |
| ICHE28d3 | 32,285,337 | 8,901,618,691 | 47.99% | 94.66% |

Note: (1) Samples: sample analysis number; (2) Clean reads: total number of pair-end Reads in Clean Data; (3) Clean bases: Clean Data total bases; (4) GC content: Clean Data GC content, that is, the percentage of G and C bases in the total base of Clean Data; (5) ≥Q30%: Clean Data The percentage of bases with a mass value greater than or equal to 30.

**Supplementary Table S3** Reads mapping information.

| Sample | Total Reads | Mapped Reads | Uniq Mapped Reads | Multiple Map Reads | Reads Map to '+' | Reads Map to '-' |
| --- | --- | --- | --- | --- | --- | --- |
| ICHC14d1 | 56,956,158 | 39,357,533 (69.10%) | 36,095,729 (63.37%) | 3,261,804 (5.73%) | 22,559,815 (39.61%) | 22,287,019 (39.13%) |
| ICHC14d2 | 58,126,840 | 39,848,759 (68.55%) | 36,479,393 (62.76%) | 3,369,366 (5.80%) | 22,955,158 (39.49%) | 22,557,412 (38.81%) |
| ICHC14d3 | 59,368,390 | 41,133,839 (69.29%) | 37,612,892 (63.36%) | 3,520,947 (5.93%) | 23,720,682 (39.96%) | 23,397,940 (39.41%) |
| ICHC28d1 | 64,359,618 | 43,299,367 (67.28%) | 39,866,882 (61.94%) | 3,432,485 (5.33%) | 24,680,215 (38.35%) | 24,313,927 (37.78%) |
| ICHC28d2 | 57,949,532 | 39,379,068 (67.95%) | 36,454,536 (62.91%) | 2,924,532 (5.05%) | 22,312,603 (38.50%) | 21,959,207 (37.89%) |
| ICHC28d3 | 58,927,588 | 40,217,632 (68.25%) | 37,171,973 (63.08%) | 3,045,659 (5.17%) | 22,816,146 (38.72%) | 22,495,109 (38.17%) |
| ICHE14d1 | 61,269,586 | 43,076,829 (70.31%) | 39,749,661 (64.88%) | 3,327,168 (5.43%) | 24,510,319 (40.00%) | 24,224,887 (39.54%) |
| ICHE14d2 | 66,412,468 | 46,001,819 (69.27%) | 42,437,597 (63.90%) | 3,564,222 (5.37%) | 26,167,952 (39.40%) | 25,832,621 (38.90%) |
| ICHE14d3 | 57,550,702 | 39,410,576 (68.48%) | 36,294,838 (63.07%) | 3,115,738 (5.41%) | 22,444,465 (39.00%) | 22,217,246 (38.60%) |
| ICHE28d1 | 61,911,074 | 41,336,102 (66.77%) | 38,186,062 (61.68%) | 3,150,040 (5.09%) | 23,475,219 (37.92%) | 23,167,326 (37.42%) |
| ICHE28d2 | 67,660,212 | 45,164,458 (66.75%) | 41,777,554 (61.75%) | 3,386,904 (5.01%) | 25,613,818 (37.86%) | 25,267,658 (37.34%) |
| ICHE28d3 | 64,570,674 | 43,095,828 (66.74%) | 39,846,508 (61.71%) | 3,249,320 (5.03%) | 24,446,069 (37.86%) | 24,113,134 (37.34%) |

Note: (1) Sample: sample analysis number; (2) Total Reads: Number of Clean Reads in a single end; (3) Mapped Reads: number of Reads mapped to the reference genome and percentage of Clean Reads; (4) Uniq Mapped Reads: Number of Reads aligned to unique locations in the reference genome and percentage of Clean Reads; (5) Multiple Map Reads: The number of Reads that were compared to multiple locations of the reference genome and the percentage of Clean Reads; (6) Reads Map to '+': The number of Reads linked to the positive strand of the reference genome and the percentage of Clean Reads; (7) Reads Map to '-': The number of Reads with negative chains in the reference genome and the percentage of Clean Reads.

**Supplementary Table S4** Functional annotation and enrichment analysis of DEGs.

| DEG Set | Total | COG | GO | KEGG | KOG | NR | Pfam | Swiss-Prot | eggNOG |
| --- | --- | --- | --- | --- | --- | --- | --- | --- | --- |
| ICHC14d vs ICHE14d | 14356 | 2,776 | 12,043 | 11,944 | 8,546 | 13,993 | 11,757 | 8,157 | 13,379 |
| ICHC28d vs ICHE28d | 3432 | 563 | 2,604 | 2,700 | 1,604 | 3,322 | 2,648 | 1,716 | 3,026 |
